# Establishing a Common Nutritional Vocabulary - From Food Production to Diet

**DOI:** 10.3389/fnut.2022.928837

**Published:** 2022-06-21

**Authors:** Liliana Andrés-Hernández, Kai Blumberg, Ramona L. Walls, Damion Dooley, Ramil Mauleon, Matthew Lange, Magalie Weber, Lauren Chan, Adnan Malik, Anders Møller, Jayne Ireland, Lucia Segovia, Xuhuiqun Zhang, Britt Burton-Freeman, Paul Magelli, Andrew Schriever, Shavawn M. Forester, Lei Liu, Graham J. King

**Affiliations:** ^1^Southern Cross Plant Science, Southern Cross University, Lismore, NSW, Australia; ^2^Department of Biosystems Engineering, University of Arizona, Tucson, AZ, United States; ^3^Data Collaboration Center at the Critical Path Institute, Tucson, AZ, United States; ^4^Faculty of Health Sciences, Simon Fraser University, Burnaby, BC, Canada; ^5^International Center for Food Ontology Operability Data & Semantics (IC-FOODS), Davis, CA, United States; ^6^INRAE, UR BIA, Nantes, France; ^7^Nutrition Department, College of Public Health and Human Sciences, Oregon State University, Corvallis, OR, United States; ^8^European Bioinformatics Institute, European Molecular Biology Laboratory (EMBL-EBI), Hinxton, United Kingdom; ^9^Danish Food Informatics, Roskilde, Denmark; ^10^London School of Hygiene and Tropical Medicine, University of London, London, United Kingdom; ^11^Illinois Institute of Technology, Chicago, IL, United States; ^12^WISEcode LLC, Reno, NV, United States; ^13^Nutrient Institute LLC, a Non-profit, Reno, NV, United States; ^14^School of Biosciences, University of Nottingham, Sutton Bonington, United Kingdom

**Keywords:** dietary composition, food composition, ontologies, nutritional security, FAIR data, knowledge representation, human health

## Abstract

Informed policy and decision-making for food systems, nutritional security, and global health would benefit from standardization and comparison of food composition data, spanning production to consumption. To address this challenge, we present a formal controlled vocabulary of terms, definitions, and relationships within the Compositional Dietary Nutrition Ontology (CDNO, www.cdno.info) that enables description of nutritional attributes for material entities contributing to the human diet. We demonstrate how ongoing community development of CDNO classes can harmonize trans-disciplinary approaches for describing nutritional components from food production to diet.

## Introduction

Food production and supply systems affect human nutrition and health in personalized and global contexts (1). However, nutrition-based decisions and data are seldom integrated along the production and supply chain. This information may affect selection of cultivars and conservation of genetic resources, the management of food supply, processing and distribution, and analysis of dietary consumption patterns segmented by various demographics (2). Although various conventions exist for naming individual chemicals and physical attributes of dietary components, comparison of data and feedback within food systems is often constrained by divergence in formal definitions and classifications (3). The exchange of knowledge and operational data between domains would benefit from a consistent framework that defines nutritional and phytochemical composition, as well as other attributes of food, including their dietary role and physiological function.

Knowledge representation underpins communication, and is particularly important for sharing complex data and information within and between diverse domains such as crop biodiversity, food supply, and nutrition (4). Defining and classifying commonly understood terminology facilitates data acquisition, exchange and interoperability, where formal systems of domain-specific controlled vocabularies such as ontologies contribute to the representation and sharing of complex knowledge (5). They do this by defining terms with human readable definitions alongside machine readable relationships that facilitate the annotation, exchange, analysis, and interpretation of data (6). Establishment of clearly defined ontology classes representing domain-specific terminology is the first step to building common platforms that are of practical value to data curators and to end-users searching for relevant information. An approachable lexical representation of objects or concepts from different perspectives, that also helps reduce ambiguities in terminology for non-specialists, is particularly important for describing datasets in food supply chains (7) (Supplementary Figure 1). For instance, nutritional composition may vary depending on factors such as cultivars, cultivation systems, processing variables, food storage and preparation. Moreover, there is a need to distinguish between individual chemical components and the method by which their concentration is determined. In many standard Food Composition Tables and Databases (FCTs/ FCDBs) such information is often conflated or absent (8).

**Figure 1:**
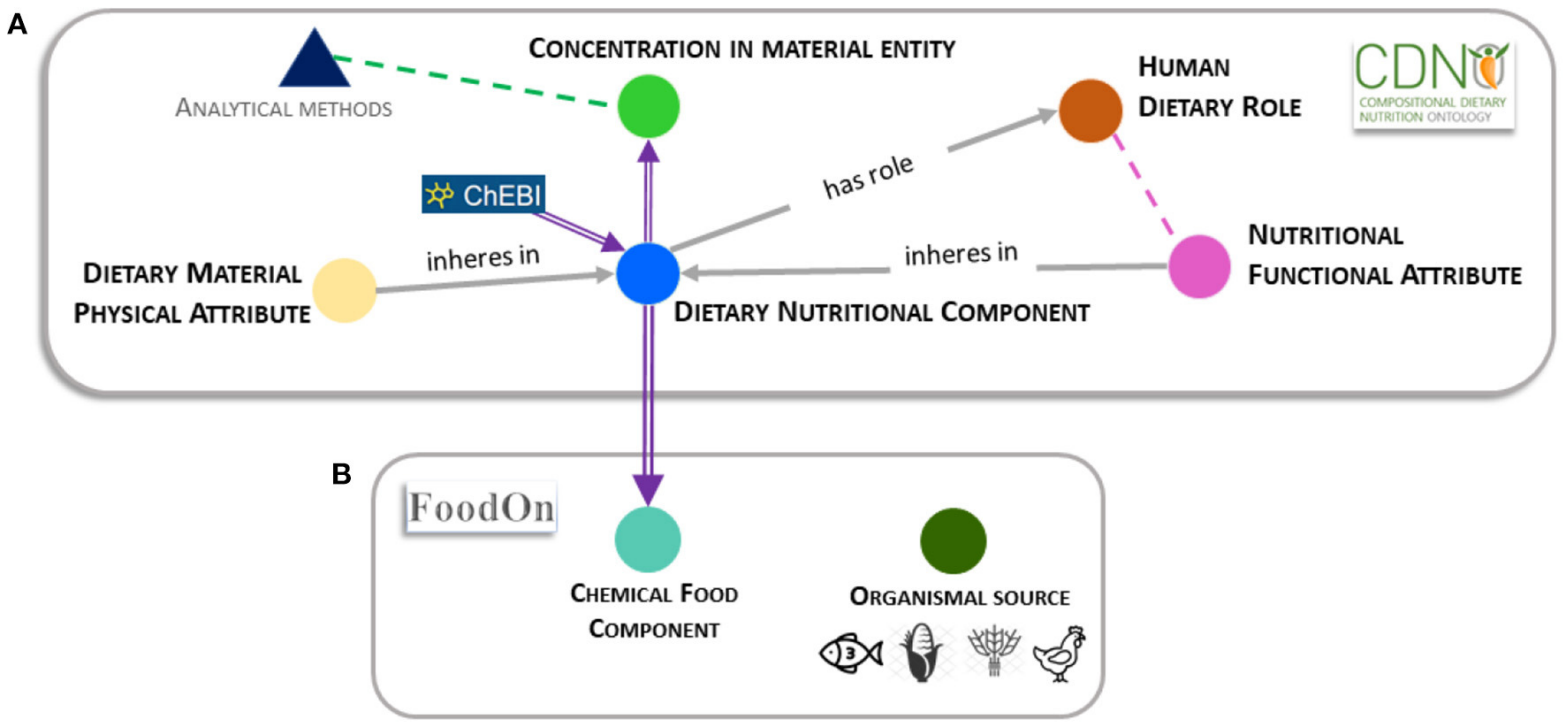
Compositional Dietary Nutrition Ontology (CDNO) class relationships and interaction with FoodON. **(A)** Relationships and associations between major ontology classes. Solid symbols (circles and triangle) represent class hierarchies that may be used individually or in combination by curators to annotate datasets in the continuum between agriculture and health. Many terms within the ‘*dietary nutritional component*’ (blue solid circle) hierarchy are imported and reused from ChEBI (purple arrow). Grey arrows indicate relationships between independent classes that may in future be adopted where evidence is available. The ‘*dietary nutritional component*’ [CDNO:0000001] provides a framework where terms are reused in the ‘*concentration of dietary nutritional component in material entity*’ [CDNO:0200001] class hierarchy (green solid circle). A distinction is made between the latter class and that required an independent ‘analytical methods' class (Figure 1, blue triangle) to provide vocabulary to describe analytical methods where terms would be used in combination to represent relevant metadata (Figure 1, green dotted arrow). The ‘*dietary material physical attribute*’ class [CDNO:0400001] (cream solid circle) provides structured subclasses to describe properties that may inhere either in a food material or be associated with a specific ‘*dietary nutritional component*’ [CDNO:0000001]. The ‘*nutritional functional attribute*’ [CDNO:0300001] class hierarchy (pink solid circle) allows the description of quantifiable functional attributes that may be associated with or inhere in terms from the ‘*dietary nutritional component*’ [CDNO:0000001] class hierarchy. Where evidence is available, terms from this class may also be associated with a human dietary role (Figure 1, pink dotted line). The ‘*human dietary role*’ [CDNO:0500001] class (orange solid circle) includes structured terms representing biological roles that may be assigned to a specific ‘*dietary nutritional component*’ [CDNO:0000001], where it is left to experts and data curators to assign supporting evidence that indicates a function defined at the levels of molecular interaction, cellular process or physiological role. **(B)** The interaction between CDNO and FoodOn is shown with a purple double arrow. FoodOn reuses ~500 terms from the CDNO ‘*dietary nutritional component*’ [CDNO:0000001] hierarchy within the ‘*chemical food component*’ [FOODON:03411041] hierarchy (cyan solid circle). The FoodOn ‘*food product by organism*’ [FOODON:00002381] class (olive solid circle) is not directly associated with CDNO classes, but can be used to describe a food source. These represent independent classes that may be combined and used in a relational, RDF or graph database by data curators to annotate and perform information extraction based on particular evidence that may require annotation.

The Open Biomedical and Biological Ontologies Foundry and Library (OBO) is responsible for the establishment and development of a wide range of formal vocabularies in the life-sciences and related domains (9). This includes the ontology for Chemical Entities of Biological Interest (ChEBI) (10), which provides a valuable resource for structured sets of chemical definitions. OBO principles emphasize the value of reusing terms (formally known as classes or properties) between ontologies. The development of the Compositional Dietary Nutrition Ontology (CDNO) (4) was prompted by the need to follow *Findable, Accessible, Interoperable*, and *Reusable* (FAIR) principles (11) of data sharing. CDNO was initially focused on vocabulary to describe nutritional components in plant-derived materials contributing to human diet, and particularly those that may vary according to crop variety or within genetic resource collections (2, 4). However, we found that the structured reusable definitions of nutritional components were equally applicable to a wide range of food raw materials derived from livestock, fish or any other organic or inorganic source described in the Food Ontology (FoodOn) (12) (Figure 1B).

## Methods

While developing and expanding CDNO, we have followed the OBO principles (13), which emphasize community development of interoperable ontologies. We focused on reuse and import of existing OBO terms, as well as ensuring open discussion within the CDNO GitHub repository (14). In order to generate terms that are subclasses of CDNO ‘*dietary nutritional component*’ [CDNO:0200001], a modified version of the Crop Dietary Nutrition Data Framework (CDN-DF) v.1.0 from Halimi et al. (15) was used, with definition and organization of additional terms arising from discussions with plant chemist domain specialists and curators from the International Network of Food Data Systems (INFOODs) collated by the Food and Agriculture Organization (FAO) (16), USDA FoodData Central (17), and the European Food Information Resource (EuroFIR) (18) food composition databases and repositories. The CDN-DF v.2.0 was used as an input for a Python script that parsed the CDN-DF_v.2.0.xlsx into the nutritional_components_framework.csv and sugar_derivatives.csv files, which were converted into input files for ROBOT templates. These templates were used to generate a revised organization of classes/terms compiled into the reference CDNO in Web Ontology Language (OWL) (19) file. Dietary nutritional components not present in the ChEBI were proposed and accepted as new entities using the ChEBI submission tool and imported into CDNO. The remaining terms that did not fit within the ChEBI scope were formally defined in CDNO, supported by reference to peer reviewed literature and authoritative online resources. These terms were described by following existing ontology definition guidelines for development of genus-differentia definitions (20). The class ‘*concentration of dietary nutritional component in material entity*’ [CDNO:0200001], as well as its subclasses were created using a Dead Simple OWL Design Pattern (DOS-DP) (21) modified from The Environment Ontology (ENVO) (22, 23). The DOS-DP combined terms from the Phenotype and Trait Ontology (PATO) (24), CDNO and the Basic Formal Ontology (BFO) (25) with OWL equivalence axioms. The remaining major classes were proposed and discussed via the CDNO GitHub issue forum (14) and in online workshops and seminars.

The CDNO ontology and accompanying code was initially created using the Ontology-Development-Kit (ODK) (26), and later versions of CDNO were developed using the templates module from the ROBOT software (27). The reference CDNO OWL file and the source code are available from Github CDNO repository (14). Additional database tables were added to the core CropStoreDB MySQL schema (28) to manage different nutritional data sources, along with an ‘ontology register' lookup table to CDNO, FoodOn, ChEBI, NCBI taxon (29) and Plant Ontology (PO) (30) terms.

## Results and Discussion

CDNO is registered as part of the OBO Foundry with terms and definitions searchable via Ontobee (31) or the Ontology Lookup Service (OLS) (32). Version 2.2 of the CDNO comprises five top level classes ‘*dietary nutritional component*’ [CDNO:0000001], ‘*concentration of dietary nutritional component in material entity*’ [CDNO:0200001], ‘*nutritional functional attribute*’ [CDNO:0300001], ‘*dietary material physical attribute*’ [CDNO:0400001], and ‘*human dietary role*’ [CDNO:0500001] (Figure 1A).

While dietary nutrients within food substrates are often present as complex and dynamic physical and chemical structures or mixtures, food labelling and FCTs/ FCDBs typically represent proximate and individual chemical components, alongside properties such as energy. Within CDNO, the primary ‘*dietary nutritional component*’ [CDNO:0000001] class is formally defined as: “A material entity taken in by an organism that contributes to the survival, growth, development, or other biological function of itself, its bionts, or its holobionts.” This class is structured with 10 subclasses corresponding to the major commonly used proximate classifications of chemical food composition, such as proteins, carbohydrates, and vitamins. The component terms are organized in an acyclic hierarchical structure with up to five additional levels. At present (v3.1) this class includes over 685 chemical terms, including 29 unique CDNO nutritional chemical components and their definitions. Within FoodOn, the ‘*dietary nutritional component*’ [CDNO:0000001] class structure has been imported as a subclass of ‘*chemical food component*’ [FOODON:03411041] class (Figure 1B). A similar hierarchical classification of dietary nutritional components that lacked ontological relationships and definitions had previously been proposed by the EuroFIR project (18, 33). The current versions of EuroFIR thesauri are available online (34). This was shared following exchange of the original CDNO framework.

Ensuring interoperability of terms defined within CDNO, along with their labels and synonyms, requires ongoing consultation with a range of specialists from different domains. The ‘*dietary nutritional component*’ [CDNO:0200001] class imports many terms defined within ChEBI, where relationships are primarily determined by formal chemical classifications. However, we were keen to establish a hierarchy that focuses on and accommodates terms organized according to sub-categories recognized by nutritionists and different domain experts such as food scientists and chemists. We generated and defined subclasses as required, and included synonyms used in different English-speaking countries. As an example, the term ‘*available carbohydrate*’ [CDNO:0000003] has the synonym “digestible carbohydrate” according to Englyst et al. (35), but should not be confused with the term “total carbohydrate” used in some food tables. The latter term is used in the USDA FCT to refer to a specific method used for carbohydrate determination, calculated by subtraction of the sum of the crude protein, total fat, moisture, and ash from the total weight of the food (36). In order to accommodate such conceptual discrepancies and reduce ambiguity, the term ‘*concentration of carbohydrate in material entity*’ [CDNO:0200005] can be used to refer to total carbohydrate, without making any assumption as to a specific type of carbohydrates.

### Context and Use of Major Classes

A major intended use of the ‘*dietary nutritional component*’ [CDNO:0000001] class is to harmonize the annotation and exchange of dietary composition datasets from a diverse range of sources that quantify concentration of chemical nutritional components (37) (Figure 1; Supplementary Figure 2). These may include data generated by analytical laboratories for production, reference and research (Figure 1A), as well as derived from existing FCTs/ FCDBs or food labelling. Such data may also be used when evaluating evidence in relation to dietary role. The ‘*concentration of dietary nutritional component in material entity*’ [CDNO:0200001] class is formally defined as: “The concentration of dietary nutritional component when measured in some material entity”. In addition, the ‘*dietary material physical attribute*’ [CDNO:0400001] class is defined as: “A physical property that inheres in a food material or one or more dietary nutritional components.” This enables a formal distinction to be made between chemical components and physical properties (or qualities) such as “potential energy” that may inhere in a food material. At present, in most FCTs/ FCDBs the tag for “energy” appears equivalent to or alongside chemical components such as sugars (Figure 1A).

We make an important distinction between the class ‘*concentration of dietary nutritional component in material entity*’ [CDNO:0200001] and terms used to describe the analytical method, by which a specific concentration is established. Diverse methods and units of measurement are associated with quantitative data in research literature, for supply chain quality assurance and control, or to inform Food Composition Tables (FCT) and labelling (Figure 1B). This requires appropriate vocabulary (potentially in an independent ‘*analytical methods*’ class) to describe the distinct steps in the process to quantify concentration (Figure 1A), including methodologies and protocols used for sampling, extraction, and analysis that may re-use terms from existing ontologies such as the Chemical Method Ontology (CHMO) (38) and the Ontology for Biomedical Investigations (OBI) (39). Many (FCTs/ FCDBs) tags conflate methods used and nutritional components measured, such as INFOODs tag names [GLYCERA] defined as “glycerides, total; determined by analysis,” [LACSM] as “lactose; expressed in monosaccharide equivalents”, or [CHOT] “carbohydrate, total; calculated by summation.” In future such tags could be annotated with a combination of *nutritional component* and *analytical method* term IDs.

**Figure 2:**
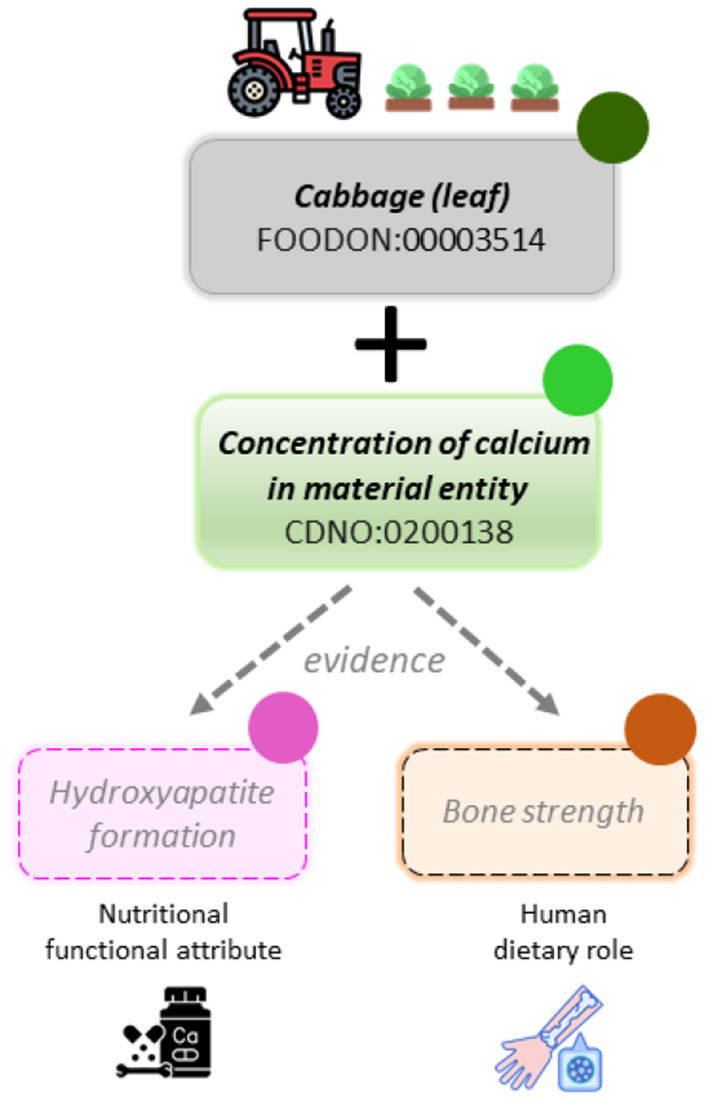
Vocabularies for annotating the food to health continuum. Schematic of proposed workflows using ontology classes to associate component concentration with independent concepts of nutritional attribute and dietary role. In data curation, each assignment requires identification of supporting evidence. Adoption of common vocabularies in diverse data repositories would facilitate data mining and inference. The FoodOn *organismal source* (olive solid circle) is used to filter available datasets, alongside the *nutritional component* terms (green solid circle). The structured vocabulary and definitions within the ‘*nutritional functional attribute'* class (pink solid circle) and the ‘*human dietary role'* class (orange solid circle), will then be available to represent concepts associated with one or more nutritional components, where a domain specialist has identified sufficient supporting evidence. These terms may be mapped and reused from existing OBO ontologies such as: the Experimental Factor Ontology (EFO) (42), the Human Phenotype Ontology (HP) (43), the Ontology for Biomedical Investigations (OBI) and the Ontology of Biological Attributes (OBA) (44).

The CDNO team has recently received positive feedback for the classification and description of additional terms to address the increasing clinical, consumer and market interest in the relationship between the nutritional composition of food and ingredients, its provenance and its ability to affect personal and public health outcomes. There is a growing but dispersed evidence base associated with “functional foods” and “nutraceuticals.” We therefore sought to provide a clear framework that distinguishes between chemical components and physical properties of material entities, the functional attributes they may possess, and any proposed associated human dietary role (Figures 1 and 2B). The CDNO ‘*nutritional functional attribute*’ [CDNO:0300001] class hierarchy (Figure 1A) is therefore defined as: “A functional attribute that inheres in one or more dietary nutritional components (or food material) and may contribute to a dietary role.” This provides a structured vocabulary that allows description of quantifiable knowledge (Figure 1A), with terms such as “antioxidant status” or “glycemic index.”

In order to represent the distinct concepts relating to potential role in the context of health and wellbeing, we then defined the ‘*human dietary role'* [CDNO:0500001] class as: “A biological role that may be assigned to a dietary nutritional component based on evidence, supported at the levels of molecular interaction, cellular process or physiological role.” The value of establishing these distinct classes is demonstrated by the ambiguity associated with use of the word “vitamin,” which may refer either to a role [CHEBI:33229] and/or to a chemical entity [CDNO:0000014], depending on context. It is recognized that any conjecture made in relation to role [BFO:0000023] is dependent on an evidence base (Figure 2B), and so the terms defined within the ‘*human dietary role'* class hierarchy are made available primarily for data curators and specialists to associate or annotate with evidence-based datasets. Moreover, a role may be dependent on many variables, including but not limited to concentration (dose), physical form (bioavailability), demographic, genetic, developmental stage and/or health status of the human subject, as well as intake of other dietary components. Such variables may be defined within other OBO ontologies such as the Ontology for Nutritional Studies (ONS) (40), which provides a framework for evidence-based studies structured according to various parameters or the Environmental Conditions, Treatments, and Exposures Ontology (ECTO) which supports modelling of exposure processes such as dietary exposures (41). We anticipate that further development of the CDNO classes described above may benefit from reuse of terms from additional OBO ontologies such as the Experimental Factor Ontology (EFO) (42), the Human Phenotype Ontology (HP) (43), OBI, and the Ontology of Biological Attributes (OBA) (44). However, substantial work between domain experts and OBO ontology curators will be required to resolve any discrepancies and allocate appropriate terms to the ‘*nutritional functional attribute*’ or ‘*human dietary role*’ classes (Figure 2).

### CDNO for Data Curation and Retrieval

The CDNO is a live open-source project that encourages regular discussion and enhancements to be proposed by stakeholders, in the food supply and nutritional domain. Current updates by the CDNO developers are made in consultation with the OBO community. The value of a common vocabulary is demonstrated by the ease with which specific terms may be associated with distinct data sources. Since CDNO is expected to facilitate the compilation and analysis of a diverse range of datasets, we present a use case interface that demonstrates how variation in the concentration of nutritional components may be compared between datasets from different sources and levels of abstraction (Supplementary Figure 2).

Analytical samples may be derived from any stage in the characterization of biodiversity, plant breeding, cultivar improvement and deployment, through the food production, processing and supply chains, as well as from food storage, preparation, consumption and digestion (Supplementary Figure 1; Figure 1A). In many cases, the ‘*concentration of dietary nutritional component in material entity*’ [CDNO:0200001] terms would be used to annotate data in a curation pipeline in conjunction with FoodOn terms that define organismal source (e.g., from crops, livestock, fisheries) and organismal part (e.g., grain, liver, fin) (Figure 1B).

In order to demonstrate a use-case we curated data that used a series of metadata terms to describe specific sets of nutritional component concentration data. This included the FoodOn ‘*Food product by organism'* [FOODON:00002381] class (Figure 1), and the National Center for Biotechnology Information (NCBI) organismal classification ontology (NCBITaxon) entity (Supplementary Figure 2). We then developed an online data retrieval interface that allows selection of specific CDNO terms to filter and access multiple sources of nutritional data derived from a crop biodiversity database (45), a national food composition database (46) and a geo-spatial dietary nutrition study (47) (Supplementary Figure 2).

One valuable outcome of generating an ontology is the opportunity to include structured annotation within the reference OWL file. This may include authoritative citations, as well as formal cross-references to published databases and other reference data sources. We therefore incorporated provisional cross-references between specific terms and widely used FCT/FCDB tagnames/code numbers. For example, the term ‘*concentration of proline in material entity*’ [CDNO:0200062] was associated with the tagname “PRO” from INFOODs and the code “1226” from USDA. Ongoing maintenance of this feature will facilitate the harmonization of vocabularies used in different FCDBs.

## Conclusion

CDNO is a new, open source, community developed, web-accessible vocabulary providing a formal representation of commonly used dietary and nutritional terminology. Ongoing development of this ontology (48) will contribute toward data sharing and interoperability, particularly for initiatives where a wide range of foodstuffs are analyzed to diversify diet and agricultural production (49). We anticipate that extending the range and harmonization of terminologies in food systems will facilitate the sourcing and management of nutritional resources, and stimulate development of information-led markets (50, 51). In particular, there is scope for large-scale data integration that enables downstream meta-analyses to complement advances in human, crop and livestock genomics and high throughput analytical chemistry.

## Data Availability Statement

The datasets presented in this study can be found in online repositories. The names of the repository/repositories and accession number(s) can be found at: https://github.com/Southern-Cross-Plant-Science/cdno.

## Author Contributions

LA-H, KB, RW, DD, RM, and GK were members of the working group that conceptualized and design of the CDNO. LA-H, KB, RW, DD, RM, ML, MW, LC, AMa, AMø, JI, LS, XZ, BB-F, PM, SF, AS, LL, and GK provided significant analysis and interpretation of research data. LA-H and GK wrote the first draft of the manuscript. KB and LA-H created the codebase for the CDNO. All authors provided critical revisions on manuscript drafts and approved the final manuscript.

## Funding

LA-H performed this work as part of a Scholarship from Southern Cross University and Crops For the Future, with additional and subsequent post-doc funding from WISEcode LLC.

## Conflict of Interest

PM and AS are employed by WISECode LLC. This study received funding from WISECode LLC. The funder had the following involvement with the study: provision of domain-specific know-how in establishing scope of the ontology and classes, along with key insights into practical end use in data management. SF was employed by Nutrient Institute LLC. The remaining authors declare that the research was conducted in the absence of any commercial or financial relationships that could be construed as a potential conflict of interest.

## Publisher's Note

All claims expressed in this article are solely those of the authors and do not necessarily represent those of their affiliated organizations, or those of the publisher, the editors and the reviewers. Any product that may be evaluated in this article, or claim that may be made by its manufacturer, is not guaranteed or endorsed by the publisher.

## Supplementary Material

Supplementary Figure 1 Representation of nutritional information in the continuum between agriculture and health. Knowledge is represented in the context of different domains that may contribute to variation in nutritional outcomes. The symbols under each domain correspond to each of the major CDNO and FoodOn classes represented in Figure 1.

Supplementary Figure 2 Use-case of CDNO terms used to mine and compare data from diverse sources. Online graphical interface developed to demonstrate how the ‘*concentration of dietary nutritional component in material entity*’ [CDNO:0200001] classes are associated with quantitative data derived from (i) genetic resources, (ii) food composition tables and (iii) a geo-spatial study. Data associated with distinct data sources (see below) have been curated within a relational schema to enable semantic search and filtering based on annotation of key records, where ontology terms are managed within an ‘ontology register' table. The user may navigate the hierarchical class tree (here, CDNO v3.0) and select (green solid circle) a nutritional component (e.g. CDNO:0200138 - ‘calcium concentration'). Food or crop groups may be selected (olive solid circle) and then refined to e.g. specific crops or organismal parts used for food. Data from the filtered datasets may be represented by a combination of box-whisker plots, frequency distributions, or single values. Calcium concentration data were sourced to represent variation within (i) a crop biodiversity collection representing the vegetable *Brassica oleracea* genepool (52), the Brassica Information Portal (45) (ii) vegetables and vegetable products from a food composition database - the Canadian Nutrient File (53) - cited in (46) and (iii) a geo-spatial study where crop-based edible food items were sampled from multiple locations in Malawi, with data presented for variation in the underutilized crop *Moringa oleifera* (47). Recorded values have been adjusted to enable direct comparison on a consistent y-axis, as the original units varied according to each study (i) %, (ii) mg/100g, (iii) mg per kg. For (ii), as for many food composition databases, only single values per nutrient component per food are available (15), although the number of original samples from which the mean value is derived is stated.
