# Supplementary figures and images for: Establishing a Common Nutritional Vocabulary - From Food Production to Diet
Source: Front Nutr. 2022 Jun 21;9:928837. doi: 10.3389/fnut.2022.928837 (PMC9265659; doi:10.3389/fnut.2022.928837)

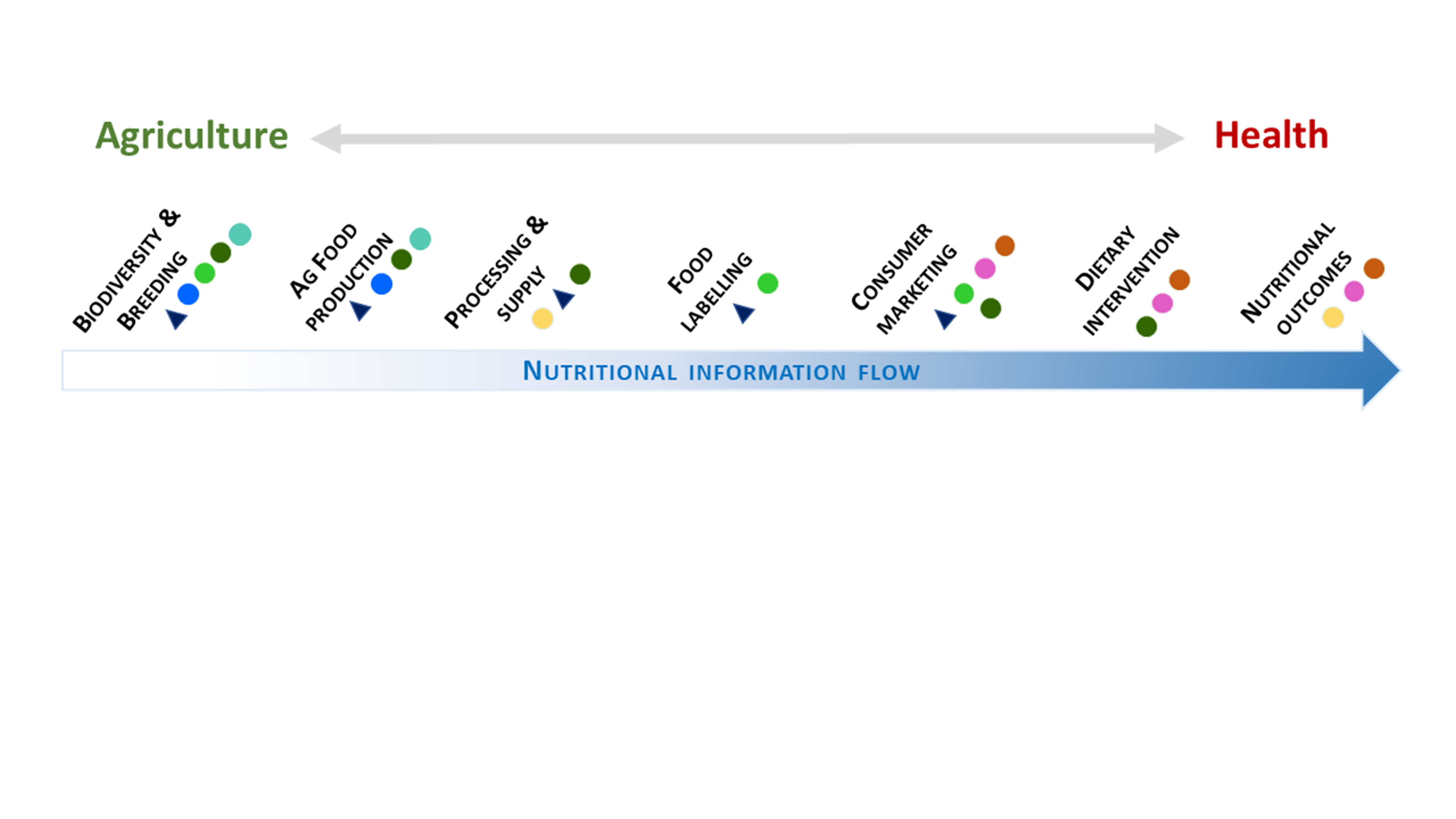

Supplement: Supplementary Figure 1 — Representation of nutritional information in the continuum between agriculture and health. Knowledge is represented in the context of different domains that may contribute to variation in nutritional outcomes. The symbols under each domain correspond to each of the major CDNO and FoodOn classes represented in Figure 1. [file Image_1.TIF]

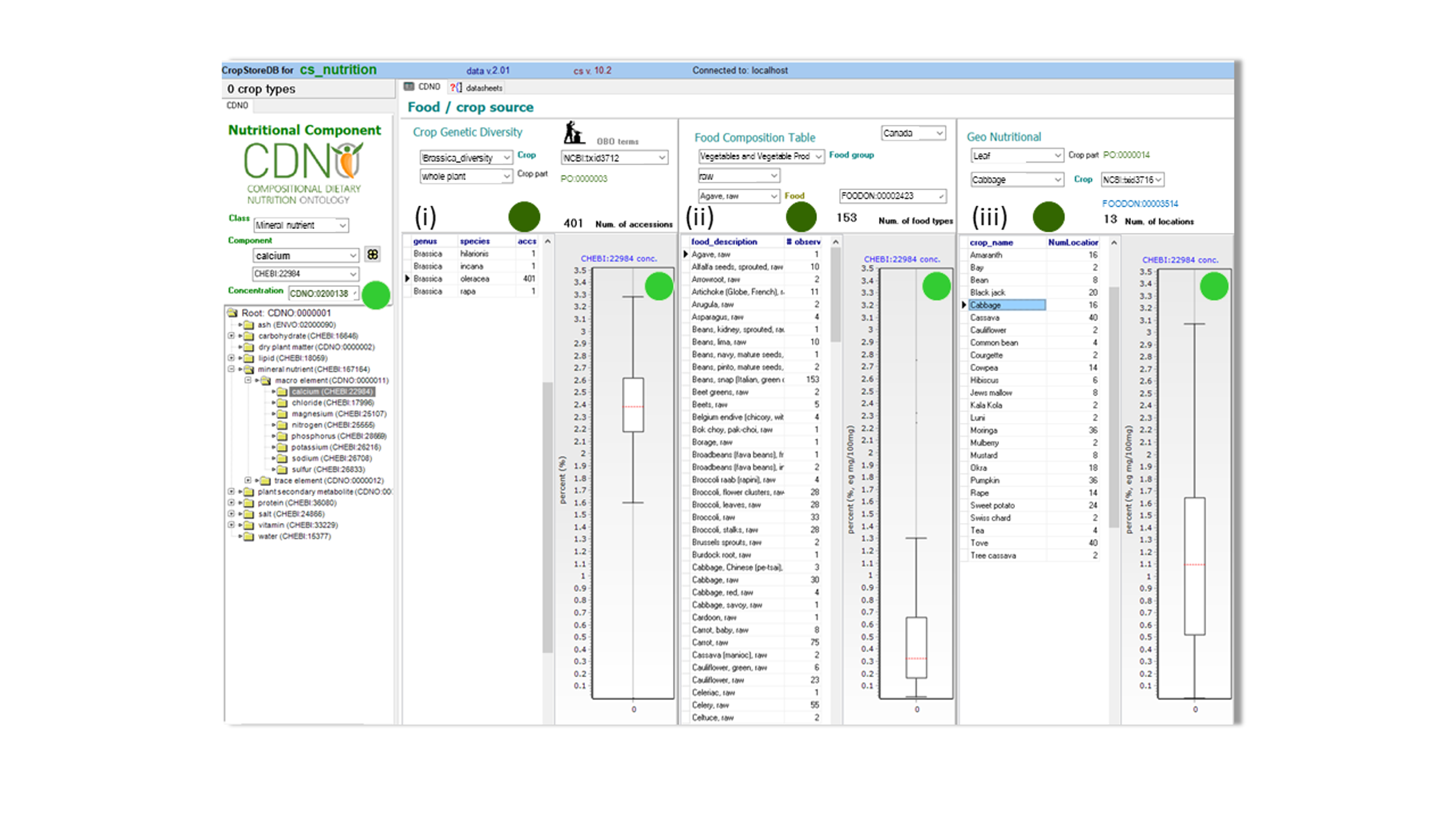

Supplement: Supplementary Figure 2 — Use-case of CDNO terms used to mine and compare data from diverse sources. Online graphical interface developed to demonstrate how the ‘concentration of dietary nutritional component in material entity’ [CDNO:0200001] classes are associated with quantitative data derived from (i) genetic resources, (ii) food composition tables and (iii) a geo-spatial study. Data associated with distinct data sources (see below) have been curated within a relational schema to enable semantic search and filtering based on annotation of key records, where ontology terms are managed within an ‘ontology register' table. The user may navigate the hierarchical class tree (here, CDNO v3.0) and select (green solid circle) a nutritional component (e.g. CDNO:0200138 - ‘calcium concentration'). Food or crop groups may be selected (olive solid circle) and then refined to e.g. specific crops or organismal parts used for food. Data from the filtered datasets may be represented by a combination of box-whisker plots, frequency distributions, or single values. Calcium concentration data were sourced to represent variation within (i) a crop biodiversity collection representing the vegetable Brassica oleracea genepool (52), the Brassica Information Portal (45) (ii) vegetables and vegetable products from a food composition database - the Canadian Nutrient File (53) - cited in (46) and (iii) a geo-spatial study where crop-based edible food items were sampled from multiple locations in Malawi, with data presented for variation in the underutilized crop Moringa oleifera (47). Recorded values have been adjusted to enable direct comparison on a consistent y-axis, as the original units varied according to each study (i) %, (ii) mg/100g, (iii) mg per kg. For (ii), as for many food composition databases, only single values per nutrient component per food are available (15), although the number of original samples from which the mean value is derived is stated. [file Image_2.TIF]
